# Safety of SGLT2 inhibitors in chronic kidney disease patients during Ramadan fasting: a prospective cohort study

**DOI:** 10.1007/s40620-025-02438-8

**Published:** 2025-10-24

**Authors:** Ahmed Elkeraie, Mohammed Elraggal, Merna AbouKhatwa, Mariam E. Omar, Rowan Zyada

**Affiliations:** 1 Nephrology Department, Faculty of Medicine, Alexandria University, Alexandria, Egypt; 2 Nephrology Department, Kidney and Urology Center, Alexandria, Egypt; 3 Nephrology Department, Johns Hopkins Aramco Healthcare, Dhahran, Saudi Arabia; 4 Department of Clinical Pharmacy and Pharmacy Practice, Faculty of Pharmacy, Alexandria University, Alexandria, Egypt; 5 Nephrology Department, EL-Qabbary Hospital, Alexandria, Egypt

**Keywords:** Fasting, Chronic kidney disease, SGLT2 inhibitors

## Abstract

**Background:**

The safety of sodium-glucose co-transporter 2 inhibitors (SGLT2i) during fasting in patients with chronic kidney disease (CKD) remains underexplored. This study investigates the risk of acute kidney injury (AKI) in patients with CKD who are fasting and taking SGLT2i, and also examines the long-term estimated glomerular filtration rate (eGFR) outcomes over 6 months after fasting.

**Methods:**

In this prospective cohort study conducted at the Kidney and Urology Centre, Alexandria, Egypt, 236 Muslim patients with CKD were enrolled during Ramadan. Patients were stratified into two groups: SGLT2i users (*n* = 56) and non-users (*n* = 180). Weekly serum creatinine and eGFR were monitored during Ramadan, with monthly follow-up for 6 months post-fasting. AKI was defined as an increase in serum creatinine ≥ 0.3 mg/dL. Logistic regression was used to identify AKI predictors.

**Results:**

The incidence of AKI was 17.8% among SGLT2i users and 23.3% among non-users (RR 0.76; 95% CI 0.41–1.42; *p* = 0.38), indicating no significant difference. No significant changes were observed in eGFR between the two groups at the end of Ramadan or during the 6-month follow-up. AKI incidence significantly increased with CKD severity (*p* = 0.036), with 37.5% of CKD stage 5 patients experiencing at least one episode. Logistic regression identified older age and lower baseline eGFR as significant predictors of developing AKI; SGLT2i use was not independently associated with the risk of developing an AKI episode.

**Conclusion:**

SGLT2i use in CKD patients during Ramadan fasting does not increase the risk of AKI. These findings may offer valuable guidance for clinicians managing fasting patients with CKD on SGLT2i therapy.

**Graphical abstract:**

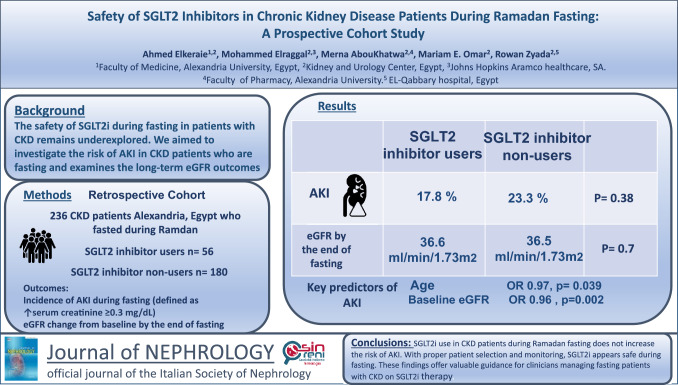

**Supplementary Information:**

The online version contains supplementary material available at 10.1007/s40620-025-02438-8.

## Introduction

Ramadan fasting, one of the five fundamental pillars of Islam, involves abstaining from food and drink from dawn to sunset for approximately 30 days every year [1]. This period is associated with substantial changes in dietary habits and in sleep patterns including prolongation of the awakening hours during the night [2]. In addition, several potential physiological challenges to the human body, including the kidney are encountered [3, 4]. The lunar cycle, which lasts 355 days per year, is the basis followed by the Islamic calendar. Hence, Ramadan, the ninth lunar month of the Islamic calendar, can fall in varying seasons and climates [1] leading to fasting duration ranging from 12–14 h, but in some countries it may extend up to 22 h depending on geographical location [5].

While specific populations, such as prepubertal children, women during their menstrual period or postpartum, long distance travellers, pregnant or breastfeeding women, elderly individuals who cannot tolerate fasting, the mentally disabled and patients with chronic diseases, including chronic kidney disease (CKD) are exempted from this religious duty, based on Islamic principles [6, 7] many still prefer to carry out this religious duty [8].

CKD is a major global health burden, and has been reported by the Global Burden of Disease (GBD) studies to be one of the predominant causes of death worldwide [9]. In 2013, CKD represented the 19th cause of mortality worldwide, rising to the 13th and 12th positions in 2016 and 2017, respectively, and is projected to become the fifth leading cause of death by 2040 [10].

The substantially increased burden of CKD has been challenging to both developing and developed countries [11]. A meta-analysis revealed that CKD affects approximately 13.4% of the general population worldwide, representing a bona fide global health and economic burden [12].

The impact of Ramadan fasting on patients with CKD is not very well understood, and nephrologists struggle to provide comprehensive evidence-based guidance for CKD patients who prefer to fast [13]. The existing literature is controversial, and no definitive guidelines or standardized protocols exist for fasting among CKD patients. Several factors contribute to this controversy, including patient age, duration of fasting, presence of other comorbidities, CKD stage, guidance offered by other healthcare providers like diabetologists and cardiologists, and the design of the existing studies [3, 14]. In addition, the focus of the previously published studies was mainly on the acute renal hemodynamic alterations that occur during fasting, while the long-term effect of Ramadan fasting on kidney function remains unclear [3, 4].

Sodium glucose co-transporter 2 inhibitors (SGLT2i) are currently considered a cornerstone in the management of CKD, together with renin angiotensin aldosterone blockers (RAAS blockers). However, it is not known whether the hemodynamic effects exerted by SGLT2i affect patients with CKD during fasting or not, especially, when combined with RAAS blockers or diuretics. Therefore, there is a compelling need for evidence-based data to guide the advice for fasting patients with CKD who are on SGLT2i.

This study investigates whether SGLT2i use during Ramadan fasting increases the risk of acute kidney injury (AKI) in patients with CKD.

## Methods

This prospective cohort study was conducted at the Kidney and Urology Centre, Alexandria, Egypt. Eligible participants included Muslim adults with CKD and stable kidney function during the 3 months prior to Ramadan. Exclusion criteria included AKI, active infection, sepsis or malignancy, and heart failure with reduced ejection fraction (HFrEF), as those patients were advised not to fast. Fasting adherence was confirmed via patient self-report at each visit.

Participants were divided into two groups: those taking SGLT2i for at least 3 months prior to Ramadan and those not taking SGLT2i. All patients received counselling on fluid intake (targeting 3 L in fast-breaking hours, as tolerable) and avoiding heat exposure. Patients with heart failure and those maintained on diuretic therapy were instructed to monitor their daily weight and were educated to adjust their diuretic dose based on weight changes. Also, patients were advised to take the diuretics at sunset to avoid being dehydrated with fasting. Serum creatinine and estimated glomerular filtration rate (eGFR) (by CKD-EPI) were monitored weekly during Ramadan and monthly for 6 months post-fasting.

Any patient with a rise in serum creatinine ≥ 0.3 mg/dl (i.e. AKI definition) was advised to discontinue fasting and follow up their kidney function tests after 1 week. Patients with stable serum creatinine or who had a rise in serum creatinine of 0.2 mg/dl or less, were advised to continue fasting with weekly monitoring. All relevant patient data were collected from medical records, including demographics, medical history, and drug history (mainly the use of diuretics, RAAS blockers and SGLT2i).

### Outcomes

The primary outcome was the incidence of AKI (defined as a rise in serum creatinine ≥ 0.3 mg/dL) among both groups at any time during the whole month of Ramadan. Secondary outcomes included eGFR trend at 6 months post-fasting.

### Statistical analysis

Statistical analyses were performed using SPSS v21.0. Normality was tested using the Shapiro–Wilk test. Between-group comparisons employed Student’s *t* test or Mann–Whitney *U* test for continuous variables and Chi-square test for categorical variables. Wilcoxon signed-rank test was used for paired non-parametric data. Multivariate logistic regression was conducted to identify predictors of AKI.

We pre-specified key variables based on clinical relevance and selected factors known to influence AKI risk in the CKD population, namely age, sex, baseline eGFR, diabetes status, CKD stage, RAAS-blocker use, diuretic use, and SGLT2i exposure.

Each candidate predictor was first tested in a univariate logistic regression against AKI incidence. Variables with a p-value < 0.10 were considered for entry into the multivariable model.

## Results

Among 236 CKD patients who fasted during Ramadan, 56 patients (23.7%) had been taking SGLT2i for more than three months before Ramadan, and 180 patients (76.3%) were not taking SGLT2i. Table 1 shows the baseline characteristics of both groups. SGLT2i users were significantly older and tended to have diabetes more than in non-users. They were co-prescribed RAAS blockers more than non-users (*p* < 0.005), while the non-users were more commonly prescribed calcium channel blockers (*p* = 0.014) and diuretics (*p* = 0.02). There was no significant difference in both groups regarding gender, other comorbidities, baseline eGFR, hemoglobin, urea, calcium, or albumin, however, SGLT2i non-users tended to have lower serum phosphorus levels. A significant difference was observed between both groups regarding CKD etiology (*p* = 0.009).

**Table 1:** Baseline characteristics of SGLT2i users and non-users

	SGLT2 inhibitor non-users *n* = 180 (76.3%)	SGLT2 inhibitor users *n* = 56 (23.7%)	*p* Value
Age (mean ± SD)	59.8 ± 13	65 ± 11.5	0.0119*
Gender, *n* (%)			
Male	107 (59.4%)	29(48.2%)	0.31
Female	73 (40.5%)	27 (51.8%)	
Cause of CKD, * n* (%)			
Diabetic kidney disease	50 (27.8%)	30 (53.6%)	
Hypertension	80 (44.5%)	19 (33.9%)	
Glomerulonephritis	11 (6.1%)	4 (7.1%)	
Solitary kidney	1 (0.6%)	1 (1.8%)	0.009*
ADPKD	5 (2.8%)	1 (1.8%)	
Unknown cause (CKDu)	12 (6.6%)	1 (1.8%)	
Transplantation	13 (7.3%)	0 (0%)	
Obstruction	8 (4.4%)	0 (0%)	
Comorbidities, * n* (%)			
Diabetes	59 (32.7%)	31 (44.4%)	0.002*
Hypertension	155 (87.5%)	54 (96.4%)	0.057
Cardiovascular disease	39 (21.6%)	17 (30%)	0.37
Baseline eGFR (ml/min/1.73 m^2^)	34.6 (48.3 – 24.5)	36.4 (48.15 – 25.7)	0.79
Baseline creatinine (mg/dl)	2.06 ± 0.79	1.9 ± 0.72	0.33
Baseline urea (mg/dl)	59.1 ± 23	68.8 ± 21.8	0.27
CKD stage, * n* (%)			
1	2(1.11%)	1(1.7%)	
2	18(10%)	7(12.5%)	0.33
3a	38(21.1%)	6 (10.7%)	
3b	54 (30%)	24 (42.86%)	
4	61(33.9%)	19(30.4%)	
5	5 (3.9%)	1 (1.79%)	
Hb (g/dl)	12.5 ± 1.7	12.1 ± 1.6	0.31
Urinary ACR, mg/g, median (IQR)	180 (66–795)	457 (83–1438)	0.0897
Calcium (mg/dl)	9.1 ± 0.68	9.2 ± 0.47	0.96
Phosphorus (mg/dl)	3.86	4.18	0.0138*
Albumin (g/dl)	4.03 ± 0.41	3.96 ± 0.44	0.44
RAAS blockers, * n* (%)	39(21.6%)	29 (51.8%)	0.000*
Loop diuretics, * n* (%)	45(25%)	23 (41%)	0.02*
CCB, * n* (%)	78 (43.3%)	14 (25%)	0.014*
Mineralocorticoid antagonists, * n* (%)	12 (6.7%)	7 (12.5%)	0.16
Statins, * n* (%)	55(30.6%)	23 (41.1%)	0.14

*SGLT2i* sodium-glucose co-transporter 2 inhibitors, *eGFR* estimated glomerular filtration rate, *CKD* chronic kidney disease, *Hb* hemoglobin, *ACR* albumin/creatinine ratio, *IQR* inter-quartile range, *RAAS* renin angiotensin aldosterone system, *CCB* calcium channel blockers, *ADPKD* autosomal dominant polycystic kidney disease

*Statistically significant at *p* ≤ 0.05

All patients were allowed to fast during Ramadan, although less commonly, some fasted against medical advice (especially those with CKD stages 4 and 5) and were followed up on a weekly basis.

The incidence of AKI was 17.8% (10 patients) among SGLT2i users and 23.3% (42 patients) among non-users (RR 0.76; 95% CI 0.41–1.42; *p* = 0.38), indicating no significant difference. Each AKI episode occurred in a distinct patient. No patient had more than one AKI episode. There was no significant difference in eGFR between the two groups by the end of the fasting period (*p* = 0.7). The median change in eGFR (post- – pre-eGFR) was similar between both groups (*p* = 0.21) (Table 2).

**Table 2:** Incidence of AKI among SGLT2 inhibitor users and non-users

	SGLT2 inhibitor non-users *n* = 180	SGLT2 inhibitor users *n* = 56	*p* Value	RR	95% CI
AKI episode	42 (23.3%)	10 (17.8%)	0.38	0.76	0.41–1.42
eGFR at the end of fasting (ml/min/1.73 m^2^)	36.6 (49.6–25.025)	36.5 (46.9–26.02)	0.7		
Median change in eGFR (ml/min/1.73 m^2^)	0 (2.74 to − 3.30)	− 0.36 (1.86 to − 4.97)	0.21		

*AKI* acute kidney injury, *SGLT2i* sodium-glucose co-transporter 2 inhibitors, *eGFR* estimated glomerular filtration rate

*Statistically significant at *p* ≤ 0.05

**Fig. 1:**
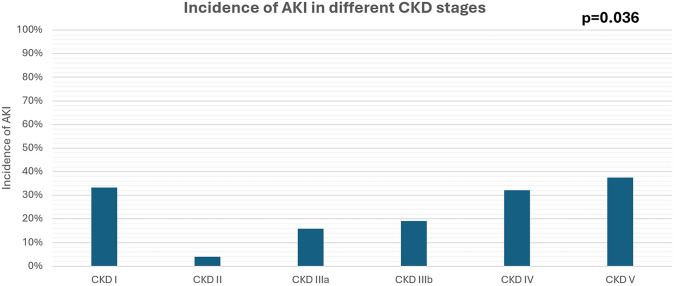
Incidence rates of AKI among CKD stages

As shown in Fig. 1, AKI incidence rates significantly differed among different CKD stages (*p* = 0.036). AKI incidence increased significantly with advanced CKD stages (*p* = 0.036). Among CKD stage 5 patients, 37.5% developed AKI, on the other hand, only 4% of CKD stage 2 patients had AKI during fasting.

By the end of Ramadan, SGLT2i users (*n* = 56) and non-users (*n* = 180) showed stable eGFR in comparison to the baseline eGFR (*p* = 0.055, *p* = 0.579 respectively) (Table 3).

**Table 3:** Comparison of pre- and post-fasting eGFR by Wilcoxon signed rank test

	Baseline eGFR (ml/min/1.73 m^2^)	eGFR at the end of fasting* (ml/min/1.73 m^2^)	*p*^1^ Value	eGFR after 6 months** (ml/min/1.73 m^2^)	*p*^2^ Value
SGLT2 inhibitor non-users	34.6 (48.3–24.5)	36.6 (49.6–25.025)	0.579	39.05 (56.07–27.7)	0.769
SGLT2 inhibitor users	36.4 (48.15–25.7)	36.5 (46.9–26.02)	0.055	39.5 (50.5–26.6)	0.650

Statistically significant at *p* ≤ 0.05

*p*^1^: Comparison between baseline eGFR and eGFR at the end of fasting

*p*^2^: Comparison between baseline eGFR and eGFR after 6 months

*Number of SGLT2 inhibitor users = 56

**Number of SGLT2 inhibitor users = 31

**Fig. 2:**
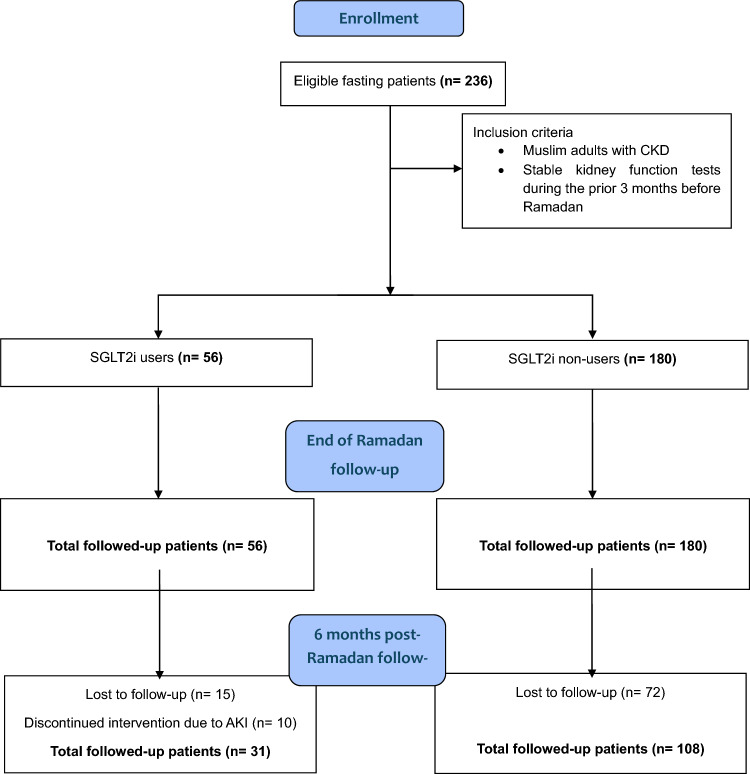
Study flow diagram

Among the whole cohort, only 139 patients were followed for the 6 months after the end of Ramadan (31 SGLT2I users and 108 non-users) (Fig. 2). No significant change was found in eGFR over time for either SGLT2 users or non-users (*p* = 0.650, *p* = 0.769, respectively) (Table 3, Figure S1).

Logistic regression analysis revealed that the incidence of AKI during fasting was significantly associated with baseline eGFR (OR 0.96, 95% CI 0.94–0.985, *p* = 0.002) and age (OR 0.97, 95% CI 0.948–0.998, *p* = 0.039) (Table 4). Further regression analysis including CKD stages did not reveal a significant difference in incidence of AKI.

**Table 4:** Multivariate regression analysis for risk factors associated with the development of AKI during fasting

Parameter	Odds ratio	95% confidence interval	*p* Value
Age	0.973	0.948–0.999	0.039*
Gender	0.627	0.321–1.223	0.17
Loop diuretics	1.267	0.571–2.786	0.56
SGLT2i	0.809	0.349–1.877	0.62
Mineralocorticoid antagonist	0.313	0.660–1.480	0.14
RAAS blockers	1.013	0.457–2.247	0.97
Baseline eGFR	0.963	0.940–0.986	0.002*

*SGLT2i* sodium-glucose co-transporter 2 inhibitors, *RAAS* renin angiotensin aldosterone system, *eGFR* estimated glomerular filtration rate

*Statistically significant at *p* ≤ 0.05

## Discussion:

Our findings suggest that SGLT2i use during Ramadan fasting does not increase the risk of AKI in CKD patients. This is particularly relevant given the rising use of these agents and the growing population of Muslim patients with CKD. Previous concerns regarding volume depletion and hemodynamic compromise were not supported by our data.

Several earlier studies evaluated kidney function changes during Ramadan in CKD patients, but most lacked any study of SGLT2i use during fasting [15]. Moreover, studies conducted on SGLT2i during Ramadan fasting included people with normal kidney function. While their results ensured the safety of SGLT2i use during fasting, they did not include patients with CKD [16–19]. A recent expert panel statement provided some guidance on the use of SGLT2i during Ramadan though without relaying specific recommendations for patients with CKD, which set the ground for our study [20].

In addition, previous studies did not include extended follow-up evaluations beyond the first one to three months after Ramadan [3, 4, 21–26]. Our study followed patients for 6 months post-Ramadan to explore the long-term effects of Ramadan fasting and possible AKI episode development on the eGFR trajectories.

Some similar studies have followed patients with CKD three months post-Ramadan. NasrAllah et al. showed that despite serum creatinine elevation in 23% of fasting patients, there was no significant difference compared to controls. The risk of eGFR decline was higher with concomitant administration of RAAS antagonists and diuretics [27]. On the other hand, a study by Bakhit et al. reported persistent worsening of kidney function after 3 months in seven out of 22 patients who experienced reduction  of kidney function during fasting. However, neither use of RAAS antagonists nor of diuretics was associated with worsening kidney function in the multivariate analysis [28].

A Turkish study published in 2023 on CKD patients found that there was no significant eGFR deterioration during fasting or 6 months post-Ramadan. Moreover, the use of RAAS antagonists did not affect eGFR [13]. None of the above-mentioned studies discussed the impact of SGLT2i use.

The higher co-prescription rate of RAAS blockers among SGLT2i users may reflect physician selection bias favoring combined therapy in patients with diabetic kidney disease with proteinuria. This is evident in the baseline median urinary ACR which was higher among SGLT2i users, despite being statistically insignificant.

Logistic regression in our cohort indicated that older age and lower baseline eGFR were the most significant predictors of AKI. These findings align with prior studies that reported similar associations. Other factors such as SGLT2i, RAAS blockers, and diuretic use were not independently predictive.

 In keeping with our findings, advanced age was identified as the sole predictor of  ≥ 25% eGFR reduction post-Ramadan within the fasting group in a study by Kara et al. [29]. On the contrary, in the study by Islam M., the regression analysis found that age did not influence the change in eGFR after Ramadan fasting [13].

Other studies highlighted different factors that may contribute to AKI while fasting. One study showed that the most significant risk factors for AKI included hypertension, a history of AKI, and liver cirrhosis [24]. Furthermore, the duration of fasting and hypertension were identified as predictors of AKI in another study [30].

Our results support the safe use of SGLT2i in CKD patients during Ramadan, provided appropriate monitoring and hydration strategies are in place. This holds especially true in patients with reduced eGFR and older age who are at higher risk of AKI. These findings may help nephrologists develop tailored guidance for fasting patients on SGLT2i therapy.

This was a single-center observational study, which limits the generalizability of the findings. Potential confounding variables could not be fully controlled, including why non-users were not prescribed SGLT2i. We did not assess acidosis or ketosis, which could have provided insights into the risk of euglycemic ketoacidosis in SGLT2i users during fasting, however, there were no reported cases of hospitalization for euglycemic ketoacidosis. Moreover, since Ramadan falls during different months each year, climate variation (e.g., higher temperatures during summer) could influence hydration status and AKI risk. The small sample size of SGLT2i users is also an additional limitation. Therefore, larger, multi-center prospective studies are needed to validate these results. Longitudinal studies would also help clarify the long-term impact of repeated fasting periods on kidney health in CKD patients using SGLT2i.

## Conclusion

In patients with CKD who choose to fast during Ramadan, the use of SGLT2 inhibitors was not found to be associated with a higher incidence of AKI. With appropriate patient selection and follow-up, SGLT2i therapy appears safe during fasting.

## Supplementary Information

Below is the link to the electronic supplementary material.

Supplementary file1 (DOCX 36 KB)

## Declarations

### Data availability statement

The data supporting the findings are available within the article and its supplementary material. Raw data of the study are available from the corresponding author upon reasonable request.
