# Supplementary material for: Safety of SGLT2 inhibitors in chronic kidney disease patients during Ramadan fasting: a prospective cohort study
Source: J Nephrol. 2025 Oct 24;38(9):2901–8. doi: 10.1007/s40620-025-02438-8 (PMC12711977; doi:10.1007/s40620-025-02438-8)
Supplement: Supplementary file 1 — Supplementary file1 (DOCX 36 KB) [file 40620_2025_2438_MOESM1_ESM.docx]

Figure S1: Change of eGFR over time in SGLT2i users and non-users

SGLT2i: Sodium-Glucose co-transporter 2 inhibitors, eGFR: estimated glomerular filtration rate, CKD: chronic kidney disease
